# Methods for Quantifying Neurotransmitter Dynamics in the Living Brain With PET Imaging

**DOI:** 10.3389/fphys.2020.00792

**Published:** 2020-07-21

**Authors:** Jenny Ceccarini, Heather Liu, Koen Van Laere, Evan D. Morris, Christin Y. Sander

**Affiliations:** ^1^Division of Nuclear Medicine, University Hospitals Leuven, Leuven, Belgium; ^2^Department of Imaging and Pathology, KU Leuven, Leuven, Belgium; ^3^Department of Biomedical Engineering, Yale University, New Haven, CT, United States; ^4^Department of Radiology and Biomedical Imaging, Yale University, New Haven, CT, United States; ^5^Department of Psychiatry, Yale University, New Haven, CT, United States; ^6^Invicro LLC, New Haven, CT, United States; ^7^Athinoula A. Martinos Center for Biomedical Imaging, Department of Radiology, Massachusetts General Hospital, Charlestown, MA, United States; ^8^Harvard Medical School, Boston, MA, United States

**Keywords:** brain imaging quantification, neurotransmitter release, kinetic modeling, dopamine, PET/fMRI

## Abstract

Positron emission tomography (PET) neuroimaging in neuropsychiatry is a powerful tool for the quantification of molecular brain targets to characterize disease, assess disease subtype differences, evaluate short- and long-term effects of treatments, or even to measure neurotransmitter levels in healthy and psychiatric conditions. In this work, we present different methodological approaches (*time-invariant model*s and *models with time-varying terms*) that have been used to measure dynamic changes in neurotransmitter levels induced by pharmacological or behavioral challenges in humans. The developments and potential use of hybrid PET/magnetic resonance imaging (MRI) for neurotransmission brain research will also be highlighted.

## Introduction

Many transformative therapies for neurological and psychiatric disease states over the last decades have targeted neurotransmitter systems through serotonin, dopamine, or opioid receptors and transporters. Neurotransmitters play an important role in regulating brain activity at the molecular and neurochemical level and are centrally involved in many brain functions, including, for example, cognition, behavior, sleep, appetite, and mood. Endogenous and exogenous stimuli, including behaviorally relevant stimuli, mood changes, and pharmacological challenges, evoke widespread changes in neurotransmitter systems. These changes are important to understand neural function in health and disease. One of the most and extensively characterized neurotransmitter system is the mesocorticolimbic dopaminergic pathway. Dopamine, both the “*pleasure”* and “*goal-directed movement”* chemical neurotransmitter, has been the main central pathway target for the pharmacological effects of habit-forming drugs. For example, dynamic changes in the dopaminergic system are known to contribute to a wide range of behaviors including affect, reward, decision-making, and inhibitory control.

Using *in vivo* functional molecular positron emission tomography (PET) imaging, we are able to image and quantify with very high sensitivity and specificity the local concentration of a range of neuroreceptor targets in a non-invasive way. PET radiotracers for imaging of neurotransmission had been primarily focused on studying changes in endogenous levels of dopamine in the striatum (Finnema et al., 2015), mainly using ^11^C-raclopride, but has not yet been adequately extended to other neurotransmitter systems (Paterson et al., 2010; da Cunha-Bang et al., 2019). More recently, advances have been made in developing new antagonist radioligands with higher dopamine D_2_ receptor affinity, such as ^11^C-FLB457 or ^18^F-fallypride and agonist radioligands like ^11^C-PHNO. Together with developments in the methodology for measuring neurotransmitter dynamics extending to extrastriatal brain regions, this has provided an increased understanding of the role of synaptic dopamine in drug action, normal neuropsychology, pathophysiology of addiction, Parkinson’s disease, and schizophrenia. Efforts in measuring neurotransmitter dynamics have currently extended to other targets such as the serotonin (Gryglewski et al., 2014; Erritzoe et al., 2019), noradrenaline, γ-aminobutyric acid, glutamate, acetylcholine, and opioid peptides [see review (Sander and Hesse, 2017)].

Data analysis methods have been developed to detect and characterize endogenous neurotransmitter release during dynamic PET imaging with a displaceable radioligand, in response to pharmacological, behavioral, or cognitive interventions, through mechanisms of a “pure competition” radioligand-target displacement model. Changes in binding potential (*BP*_*ND*_) represent receptors as static targets that are not dynamically regulated by processes like internalization within post-synaptic membranes [see Ginovart (2005) for a review of this topic]. Models that incorporate receptor internalization and its effect on PET quantification have been proposed in by integrating PET with functional magnetic resonance imaging (fMRI) studies [see section “Imaging Dynamic Neurotransmission Using Simultaneous PET and Functional MRI” and Sander et al. (2015)]. The most commonly used drug to induce dopamine change is amphetamine, exhibiting several well-documented effects on dopamine neurotransmission, including increased synthesis and release together with inhibited degradation and uptake.

Conventional PET methods to estimate the *BP*_*ND*_ are commonly based on kinetic models that assume that the system under investigation is at equilibrium. However, this assumption is intentionally violated in studies using pharmacological or behavioral stimuli to invoke transient dopamine release. When the assumption of a steady-state neurotransmitter level is violated, conventional analysis methods, which rely on time-invariant parameters (*time-invariant model*), may produce biased *BP*_*ND*_ estimates (Yoder et al., 2004; Sullivan et al., 2013). Therefore, methods to detect neurotransmitter release during an activation study have been developed allowing for a non-constant dopamine level during the scan (i.e., *time-variant parameter models*). For example, the linearized simplified reference tissue model or the linearized simplified reference region model (LSRTM or LSRRM) models dopamine release as an exponential decay that peaks instantaneously at the start of the stimulus (Alpert et al., 2003). A more flexible and innovative kinetic model to fully characterize endogenous neurotransmission, named the linear parametric neurotransmitter PET (lp-ntPET) model (Morris et al., 2005; Normandin et al., 2012; Wang et al., 2017), allows the dopamine curve to take on a variety of forms with a peak dopamine concentration to occur sometime *after* the start of the task. However, the performance of lp-ntPET remains suboptimal. It is sensitive to noise and limited in sensitivity and accuracy.

Additionally, dynamic changes in neurotransmission are also known to contribute to blood oxygenation level dependent (BOLD) and cerebral blood volume (CBV) changes (Mandeville et al., 2013). The advent of hybrid PET/MRI scanners paved the way for more comprehensive investigation of the relationship of simultaneous changes in neuroreceptor occupancy and hemodynamic parameters, therefore clarifying the contributions of specific neurotransmitter systems to dynamic changes in BOLD response (Sander et al., 2013).

In this article, we first summarize the basics of various PET methodological approaches to measure dynamic changes in endogenous neurotransmitter levels induced by pharmacological or behavioral challenges. Next, we discuss how the use of simultaneous PET and fMRI can provide complementary and new views on quantitative imaging of neurotransmission.

## Approaches for Measuring Endogenous Neurotransmitter Release: The Current Status

### Time-Invariant Models

Traditional analyses of dynamic PET quantification of changes in neurotransmitter levels estimate the *BP*_*ND*_, a static parameter that represents the potential for specific binding of the radioligand to specific enzymes in the brain, by fitting the dynamic data with compartmental or graphical (linearized) models. These models, including reference region models, such as the SRTM (Lammertsma and Hume, 1996), the Logan graphical reference method (Logan et al., 1990), and equilibrium analysis, assume that the system under investigation is in equilibrium condition. Another assumption is that the endogenous neurotransmitter and receptor concentration does not change during the course of the scan. Under these conditions, *BP*_*ND*_ is estimated from a reference tissue model considering the time activity curve of the reference region as an indirect input function to the kinetic model of the target region.

In these traditional studies, *BP*_*ND*_ is measured at rest and after a specific (cognitive or pharmacological) stimulus, during two separate PET sessions. The standard calculated endpoint to quantify neurotransmitter release is formulated as the fractional reduction in the radiotracer *BP*_*ND*_ following the stimulus (post-stimulus, *BP${}_{N\,D}^{p\,o\,s\,t}$*) compared to the *BP*_*ND*_ at rest or baseline (pre-stimulus) (*BP${}_{N\,D}^{p\,r\,e}$*), Eq. (1):

(1) $$\Delta \,B\,P_{N\,D}=\frac{\left(B\,P_{N\,D}^{p\,o\,s\,t}-B\,P_{N\,D}^{p\,r\,e}\right)}{B\,P_{N\,D}^{p\,r\,e}}$$

A decrease in *BP*_*ND*_ is used as an index of neurotransmitter release induced by a stimulus. This approach has been used conventionally with ^11^C-raclopride, ^18^F-fallypride, ^11^C-(+)-PHNO, ^11^C-FLB457, ^11^C-CIMBI-36, and ^11^C-carfentanil PET, to quantify respective endogenous dopamine (Martinez et al., 2003; Montgomery et al., 2007; Wai et al., 2019; Zakiniaeiz et al., 2019; Whitton et al., 2020), serotonin (Erritzoe et al., 2019), and opioid release (Turton et al., 2018) before and after the administration of cannabis, nicotine, methylphenidate, and amphetamine.

However, traditional models that estimate *BP*_*ND*_ do not contain explicit functions to describe short-lived neurotransmitter responses. When the stimulus is applied during a single scanning session, inconsistency in the results [of smoking studies, for example, Brody et al. (2004, 2010)] could be attributed to limitations of the conventional models. As *BP*_*ND*_ estimates become sensitive to the amount of data used post-stimulus, poor fitting of the data can lead to biased estimates of *BP* demonstrated by Sullivan et al. (2013), as critically reviewed by Liu et al. (2019). Therefore, a more flexible time-varying kinetic model would be better configured than conventional time-invariant models to reliably and reproducibly capture transient responses.

### Temporal Changes in Neurotransmitter Levels: Models With Time-Varying Terms

The first neurotransmitter competition kinetic model that was implemented to detect and characterize changes in ligand binding using only a single PET experiment is the linear extension of the reference region model (LSRTM or LSRRM), proposed by Alpert et al. (2003). LSRRM accounts for time-dependent changes in radiotracer binding, influx, and clearance induced by cognitive or drug effects in a single scan session, with the inclusion of both baseline and activation terms. LSRRM models neurotransmitter release as an exponential decay that peaks instantaneously at the start of the stimulus. It assumes that the physiologic steady state is not maintained throughout the paradigm but accounts for time-variation in the dissociation rate of ligand *k*_2_*_*a*_* = *k*_2_/[1 + *BP*_*ND*_], where *k*_2_ is the tissue-to-plasma efflux constant in the tissue region. The dopamine–radioligand competition at the receptor site is reflected by a temporal change in apparent dissociation rate, which is accounted for by adding a time-dependent parameter γ*h(t)* to a fixed *k*_2_*_*a*_*. The parameter γ represents the amplitude of the ligand displacement, hence the peak dopamine level. The function *h(t)* in Eq. (2) describes the rapid change after task onset and dissipation over time, where *u(t)* is the unit step function, while τ controls the rate at which activation effects die away and *T* indicates the timing of stimulus initiation:

(2) $$h\,\left(t\right)=e^{\left[-\tau \,\left(t-T\right)\right]}\,u\,\left(t-T\right)$$

An increased *k*_2_*_*a*_* reflects a decreased *BP*_*ND*_ for D_2_/D_3_ receptors, which in turn can be ascribed to an increased dopamine release and will result in a positive value of γ.

**FIGURE 1:**
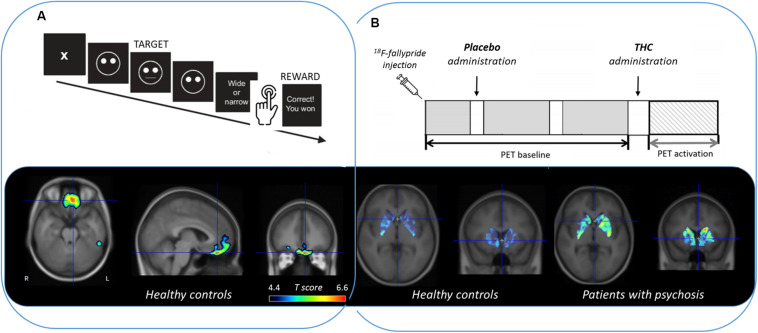
Extrastriatal and striatal dopamine release measured with ^18^F-fallypride PET and quantified with LSRRM (reported as a statistical parametric *t* map based on the significance of γ) during **(A)** a reward responsiveness learning task in healthy controls (Ceccarini et al., 2012) and **(B)** Δ^9^-THC (the main psychoactive ingredient of cannabis) administration in healthy controls compared to patients with psychosis (Kuepper et al., 2013).

There have been promising results using a single ^18^F-fallypride injection protocol and the LSRRM to describe extrastriatal and striatal dopamine release induced by emotional processing (Badgaiyan et al., 2009), attention, reward, and stress task (Christian et al., 2006; Lataster et al., 2011; Ceccarini et al., 2012; Kasanova et al., 2017), and also during dopamine-releasing pharmacological challenges, such as intravenous alcohol administration (Leurquin-Sterk et al., 2018) and Δ^9^-THC (Kuepper et al., 2013), as can be seen in Figure 1.

However, LSRRM assumes that effects of the stimuli on endogenous neurotransmitter release are instantaneous, maximal at the start time of stimulation [equal to *T*, see Eq. (2)], and decay exponentially to baseline thereafter (at rate *τ*). When any of these assumptions are violated, the estimates of the model parameters could be biased and/or inaccurate.

More flexible kinetic approaches and associated resolution models have been proposed to resolve these limitations, such as the lp-ntPET model (Morris et al., 2005; Normandin et al., 2012; Kim et al., 2014). In order to estimate the temporal characteristics of a transient neurotransmitter component in PET data with less stringent assumptions than LSRRM, lp-ntPET employs gamma-variate basis functions spanning a wide range of feasible neurotransmitter shapes, times of onset, and duration.

Specifically, lp-ntPET is the union of the conventional multilinear reference tissue model (MRTM) (Ichise et al., 2003) and a time-varying term that describes the transient neurotransmitter term, Eq. (3):

(3) $$C_{T}=R_{1}\,C_{R}\,\left(t\right)+k_{2}\,\int_{0}^{t}C_{R}\,\left(u\right)\,d\,u-k_{2\,a}\,\int_{0}^{t}C_{T}\,\left(u\right)\,d\,u-\gamma \,\int_{0}^{t}C_{T}\,\left(u\right)\,h_{i}\,\left(u\right)\,\mathrm{du}$$

(4) $$h_{i}\,\left(t\right)={\left(\frac{t-t_{D}}{t_{P}-t_{D}}\right)}^{\alpha }\,e\,x\,p\,\left(\alpha \,\left(1-\frac{t-t_{D}}{t_{P}-t_{D}}\right)\right)\,u\,\left(t-t_{D}\right)$$

The novelty and the flexibility of the lp-ntPET approach consists of the use of the, γ, gamma-variate functions *h*_*i*_(*t*), where γ describes the response magnitude, and the three implicit parameters (*t*_*D*_, *t*_*P*_, α) describe the time course of the response (*t*_*D*_, the start of the response or time delay; *t*_*P*_, the peak time; and α, the decay rate or sharpness), assuming values incremented over finite intervals. The efficiency of lp-ntPET makes it practical to perform a voxel-by-voxel analysis of the whole brain or for localized activation patterns. The model has been successfully applied to ^11^C-raclopride data to estimate the temporal dynamics of dopamine release in the mesolimbic circuit during smoking (Cosgrove et al., 2014; Kim et al., 2014). Recently, lp-ntPET has been applied in preclinical ^11^C-raclopride PET studies following amphetamine administration (Angelis et al., 2019).

To evaluate the validity of extending lp-ntPET to the cortex, Liu et al. (2018) compared the ability of the lp-ntPET model and LSRRM to detect and characterize cortical dopamine release induced by a stress task with simulated ^18^F-fallypride PET data. In ^18^F-fallypride PET studies that detect cortical DA release induced by a sustained behavioral stress challenge, simulations suggest that both LSSRM and MRTM methods produce comparable *t*-scores over a wide range of sharpness, α, and rise time for DA signals. However, LSRRM consistently outperformed MRTM in terms of fitting accuracy. This may be relevant if the study goal is to characterize the PET signal and/or to deduce the dopamine signal shape or duration. Further, LSRRM and other time-varying models may be the safer choice when the duration of the dopamine response and/or its shape are pertinent or unknown (Liu et al., 2018).

The significance of the responses estimated by lp-ntPET can be assessed using model selection criteria and statistical testing on γ, the estimated response magnitude, obtained by either *t* or *F*-test values comparing the goodness of fit of MRTM and lp-ntPET for each data set (Normandin et al., 2012). However, it has been reported that the *t*-score is not an indicator of goodness of fit but merely a measure of the magnitude of an estimated parameter relative to its variability (Liu et al., 2018). Additionally, the goodness of fit metrics depend critically on the number of parameters in the model and, together with the number of basis functions, have an impact on the false positive rate (FPR) (Liu and Morris, 2019). Given their findings, Liu and Morris (2019). proposed a set of modified goodness-of-fit metrics that adapt to the number of basis functions to maintain a stable FPR (Liu and Morris, 2020). Another recent Monte Carlo method proposed by Bevington et al. (2020) improves the detection sensitivity while preserving the cluster size threshold.

An optimization of the lp-ntPET displacement modeling method, called 2-step lp-ntPET, has been proposed by Mérida et al. (2018) where the model parameters are estimated in two steps, starting with the estimation of *R*_1_, *k*_2_, and *k*_2_*_*a*_* with MRTM followed by the estimation of the release parameters (*t*_*D*_, *t*_*P*_, α, and γ) (unpublished results). In this way, the kinetic parameters are estimated more accurately, independently from the magnitude of the endogenous neurotransmitter release, and the macroparameter, the displacement ratio, seems to allow better detection of neurotransmitter discharge.

Finally, recent developments regarding the investigation of neurotransmitter dynamics have been introduced. For instance, the assessment of temporal changes in dopamine release has been proposed to advance to a resolution of a few minutes using detailed modeling of dopamine dynamics (Lippert et al., 2019). Furthermore, the assessment of rapid changes in dopamine release and synthesis rates during cognitive performance have also been investigated by extending the technique of functional PET (fPET) imaging using ^11^C-raclopride (Zhang et al., 2019) and ^18^F-FDOPA (Hahn et al., 2019).

## Imaging Dynamic Neurotransmission Using Simultaneous PET and Functional MRI

With the current availability of scanners capable of simultaneous dynamic PET/MRI acquisitions, there is an increasing interest in measuring endogenous neurotransmitter release and time-varying measures of receptor occupancies in combination with dynamic neurovascular changes using fMRI techniques. The capability to combine multi-modal fMRI measures and neuroreceptor PET during activation paradigms provides an unprecedented opportunity to study neurotransmission dynamics through multiple lenses in the living brain. Indeed, as technical and methodological advances in simultaneous PET/MRI have matured (Ladefoged et al., 2017; Sari et al., 2017; Chen et al., 2018), we have seen an emergence of biological questions that favor simultaneous acquisitions of PET and MR signals (Cecchin et al., 2017; Streeter Barrett et al., 2019; Sander et al., 2020).

A revolution offered by PET/fMRI is the potential for resolving dynamic transitions in brain physiology, chemistry, and neurotransmission in space and time. Being able to acquire simultaneous functional measurements under the same physiological or pharmacological conditions not only reduces confounding factors, interscan, and intrasubject variability but also enables cross-validation of biological measurements. A key importance of simultaneously acquired PET/fMRI signals for imaging dynamic neurotransmission is the ability to link actions at receptors, such as ligand binding or adaptations, to changes in hemodynamics. Alterations in local hemodynamics (such as BOLD, local perfusion, or CBV) have been shown to occur in response to changes in neuronal activity and during neurotransmission (Logothetis et al., 2001; Jenkins, 2012), although the topic of neurovascular coupling is still an active research area. The opportunity to now study a new facet of neurotransmission—neurotransmitter or receptor changes and its relationship to fMRI signal—will further elucidate the nature and interpretation of fMRI. Conversely, being able to track vascular changes during PET receptor measurements can address important questions like the dependence of PET signals on radiotracer delivery. On the latter topic, it has been demonstrated that blood flow is not a confound during dynamic PET neuromodulation experiments that evaluate within-scan time-dependent challenges (Sander et al., 2019). The advent of simultaneous PET/MR scanners has paved the way for investigations that evaluate how dopamine receptor densities may organize functional cortical networks during working memory (Roffman et al., 2016) or delineate the role of opioid receptor availability while evaluating pain processing pathways (Karjalainen et al., 2017). Time-varying PET kinetic models will be a crucial component of a comprehensive investigation between functional brain organization, physiological and molecular processes within similar timescales.

Several PET/MRI studies have been performed that have investigated the effects of time-varying neurotransmitter modulations (using pharmacological challenges) on fMRI and receptor-specific PET signals simultaneously. Much of this line of work has focused on establishing contributions of the dopaminergic system on the fMRI response, demonstrating that the hemodynamic response can be directly linked to D_2_/D_3_ receptor occupancy through neurovascular coupling mechanisms (Sander et al., 2013). The relationship between hemodynamic changes (e.g., BOLD, CBV, or CBF) as measured with different fMRI techniques, receptor occupancy, and endogenous neurotransmitter has been described with a neurovascular coupling model (Sander et al., 2013, 2015). Considering specifically a ligand L (e.g., an administered drug) with efficacy ε_*L*_ that binds to D_2_ receptors and displaces dopamine (DA), a linear relationship between hemodynamic changes (Δ*H*) and receptor occupancy (θ) is expressed as:

(5) $$\text{Δ}\,H\,\left(t\right)=N_{D\,2}\,\varepsilon_{L}\,B_{m\,a\,x,D\,2}\,\theta_{L}\,\left(t\right)-N_{D\,2}\,B_{m\,a\,x,D\,2}\,\text{Δ}\,\theta_{D\,A}\,\left(t\right)$$

where *N*_*D2*_ is a neurovascular coupling constant (determined experimentally), *B*_*max,D2*_ is the total concentration of D_2_ receptors, θ_*L*_(*t*) is the time-varying occupancy of an exogenous ligand (if present), and Δθ_*DA*_(*t*) is the time-varying occupancy of dopamine. In a more general framework, the functional hemodynamic response Δ*H* can be expressed as the sum of receptor occupancies by different ligands, L, (endogenous and exogenous) and receptors, R, as:

(6) $$\text{Δ}\,H\,\left(t\right)=\sum_{R=1}^{\#\,r\,e\,c\,e\,p\,t\,o\,r\,s}\sum_{L=1}^{\#\,l\,i\,g\,a\,n\,d\,s}N_{R}\,\varepsilon_{R,L}\,B_{m\,a\,x,R}\,\text{Δ}\,\theta_{R,L}\,\left(t\right)$$

**FIGURE 2:**
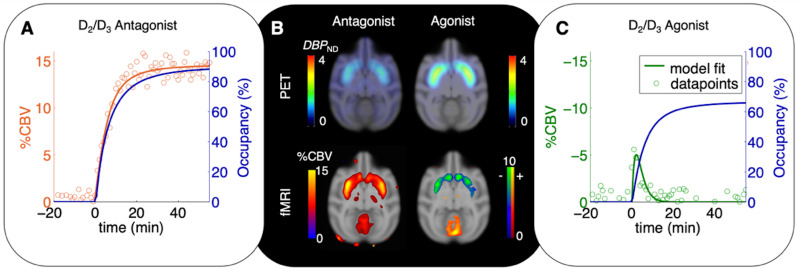
Time-varying occupancy (*blue*) and cerebral blood volume (CBV) responses [positive (*orange*) or negative (*green*) percent change] due to a pharmacological response from the D_2_/D_3_ antagonist prochlorperazine **(A)** and the D_2_/D_3_ agonist quinpirole **(C)** in non-human primates. Occupancy curves are derived from a time-varying specific binding term during kinetic modeling, and the CBV curves are fitted using the general linear model. **(B)** ^11^C-raclopride-PET binding potential maps (*upper row*) and CBV maps shown at peak value of the dynamic modeling term (Sander et al., 2015).

This model incorporates the possibility of any number of receptors and neurotransmitters (or ligands) that may contribute to the hemodynamic response. Experimental evidence for this relationship has been demonstrated using D_2_/D_3_ antagonism and agonism (Figure 2), yet remains to be evaluated for multiple receptor systems working in parallel. The temporal correlation proposed in this model can be complicated by other biological parameters like receptor desensitization and internalization *in vivo* and requires further expansion of these model frameworks (Sander et al., 2015).

The effects of a partial serotonin receptor agonist have also been evaluated using simultaneous PET/fMRI (Hansen et al., 2017), demonstrating that biphasic functional signals can be linked to serotonin receptor occupancies. As an example of stimulus-based simultaneous PET/fMRI studies, the opioid pain system has been examined using ^11^C-diprenorphine (Wey et al., 2014). In the thalamus, co-localized and positively correlated fMRI and PET signal changes suggest that opioid neurotransmission contributes to pain-induced fMRI changes.

The use of time-varying models to quantify dynamic receptor occupancy together with fMRI has been key to these types of studies. For the purpose of comparing time-dependent kinetic rate constants, outcome measures such as “dynamic binding potential” (*DBP*) have been proposed to signify the dynamic nature of the system (Sander et al., 2013). Since dynamic measurements with PET versus fMRI still operate on different time resolutions (minutes vs. seconds), the combination of time-varying outcome measures from PET kinetic models with fast-changing repetitive signals in fMRI can present a challenge for direct and equivalent comparisons in the temporal domain. As more studies are carried out in this area, careful multi-modal experimental design together with integrating multi-modal models will no doubt play a key role.

The exploration of dynamic neurotransmission with simultaneous PET/fMRI is still in its infancy. Within the field of neuropsychiatry, PET/fMRI can help evaluate whole-brain *functional* effects of antipsychotic drug treatments in relation to neurotransmitter or receptor changes (Selvaggi et al., 2019) and shed a light on distributed networks that these drugs modulate. Connecting findings from multi-modal outcomes in complex mental illness, e.g., in schizophrenia, can also serve to connect hyper- and hypo-neurotransmitter tone to cortical function during relevant cognitive tasks (Slifstein et al., 2015). These approaches extend the field beyond traditional hemodynamic-based functional imaging methods or pure pharmacological target evaluations.

## Conclusion

Quantifying dynamic neurotransmission in the living brain with PET has provided insight into the molecular dynamics of the living brain. Both time-invariant and time-varying pharmacokinetic models have played an important role in the ability to accurately quantify neurotransmitter dynamics. In the age of multi-modal methods, simultaneous PET/fMRI can have a profound impact on our understanding of neuropsychiatric diseases, drawing connections between neurotransmitter imbalances to wide-spread changes in functional activation in diseases such as addiction, psychosis, and depression. Overall, models and methods for imaging neurotransmission, non-invasively, will play an important role in elucidating mechanisms underlying brain (dys)function in health and disease.

## Author Contributions

JC and CS revised the literature and wrote the first draft of the manuscript. HL, KV, and EM revised it critically for important intellectual content. All authors contributed to the article and approved the submitted version.

## Conflict of Interest

EM is a half-time employee of the company Invicro LLC. The remaining authors declare that the research was conducted in the absence of any commercial or financial relationships that could be construed as a potential conflict of interest.
